# Damage control strategy involving early strategic decision-making and staged surgical management in a hybrid emergency room system: A case report

**DOI:** 10.1097/MD.0000000000043681

**Published:** 2025-08-01

**Authors:** Teppei Tokumaru, Takehiro Okabayashi, Yuichi Saisaka, Hideaki Kurata, Michiaki Hata, Joji Tomioka

**Affiliations:** a Department of Emergency and Critical Care Medicine, Kochi Health Sciences Center, Kochi, Japan; b Department of Gastroenterological Surgery, Kochi Health Sciences Center, Kochi, Japan; c Department of Emergency and Critical Care Medicine, Yonemori Hospital, Kagoshima, Japan.

**Keywords:** damage control strategy, early strategic decision-making, hybrid emergency room system, polytrauma, staged surgery

## Abstract

**Rationale:**

The hybrid emergency room system (HERS) has gained recognition for enabling rapid diagnosis and intervention in trauma care. However, it remains unclear whether its benefits stem solely from the availability of integrated technology or from facilitating early strategic decision-making by the trauma team.

**Patient concerns:**

A 58-year-old woman was involved in a motor vehicle collision and sustained severe injuries, including a diaphragmatic rupture, multiple left rib fractures, and thoracolumbar vertebral fractures. Despite initial resuscitation at a referring hospital, she remained hemodynamically unstable.

**Diagnoses:**

Upon arrival at our HERS-equipped facility, imaging and clinical assessment confirmed traumatic diaphragmatic rupture, flail chest, and unstable thoracolumbar vertebral fractures.

**Interventions:**

A multidisciplinary team was mobilized based on prearrival information, and an early treatment strategy was established. Emergency laparotomy was initiated 26 minutes after arrival for hemorrhage control, followed by staged rib fixation and spinal stabilization surgeries in accordance with her physiological condition.

**Outcomes:**

The patient recovered fully with no neurological deficits and was discharged in good general condition after completing all staged interventions.

**Lessons:**

This case suggests that the effectiveness of HERS lies not only in its integrated infrastructure but also in its capacity to enable early strategic decision-making and coordinated staged interventions. These principles may be adaptable to non-HERS trauma centers that emphasize early information sharing and multidisciplinary planning.

## 1. Introduction

Damage control strategies are fundamental principles in the management of severe polytrauma, where rapid decision-making and appropriate prioritization of surgical interventions are critical.^[1,2]^

The hybrid emergency room system (HERS), which integrates imaging, anesthesia induction, and emergency surgery within a single space, has recently attracted attention as a platform that may facilitate more efficient trauma care.^[3–5]^ Several studies have suggested that HERS can shorten the time to intervention and improve outcomes for critically injured patients.^[4,5]^

However, the specific factors within the HERS that most significantly contribute to improved outcomes remain unclear. Particularly, it is uncertain whether the benefits of HERS arise merely from the availability of equipment or from enhanced team-based strategic decision-making in the early phase of trauma care.

Therefore, we aimed to report a case of severe polytrauma successfully managed through early information sharing, strategic decision-making, and immediate execution of a damage control strategy within a HERS setting, followed by a staged surgical approach tailored to the patient’s physiological condition. We believe that this case was notable for the integration of early strategic planning and a staged surgical approach within a single coordinated setting, providing an opportunity to reflect on how trauma team coordination may influence outcomes.

## 2. Case presentation

**Figure 1.**
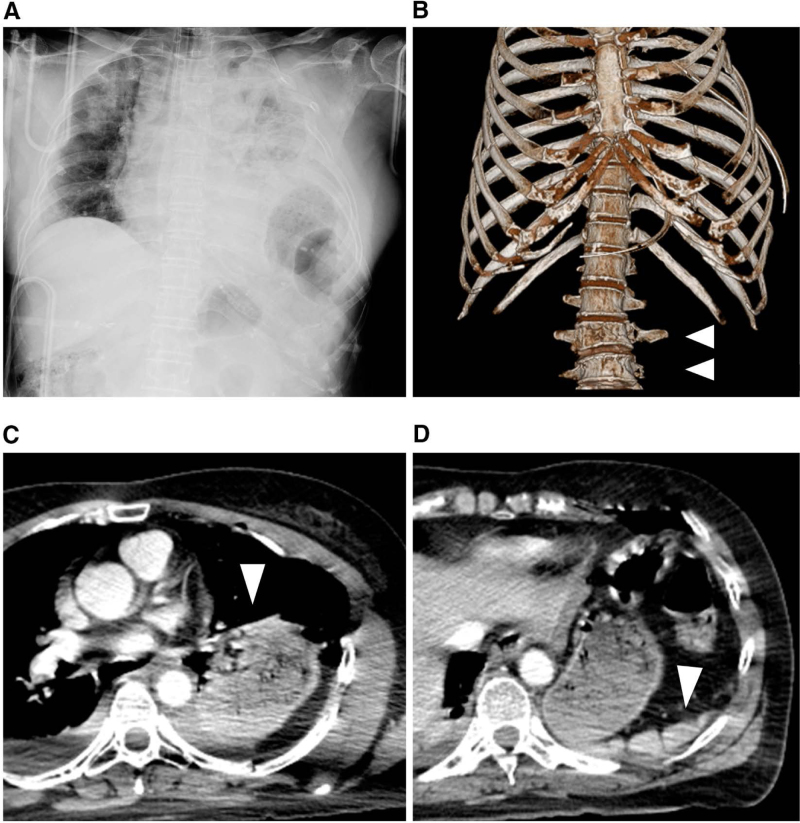
Radiographic imaging on admission. (A) Chest radiograph shows a left diaphragmatic injury with a hernia and multiple rib fractures. (B) Computed tomography (CT) shows the ruptured lumbar vertebral fractures (white arrowheads). (C, D) CT shows the left diaphragmatic injury and hernia and splenic injury (white arrowhead). The diaphragmatic injury corresponds to an AAST-OIS grade IV. AAST-OIS = American Association for the Surgery of Trauma–Organ Injury Scale, CT = computed tomography.

A 58-year-old woman, unrestrained in the rear seat of a passenger vehicle, was involved in a head-on collision at an intersection. Upon arrival at the scene, a physician-staffed rapid-response team found her in hemorrhagic shock. Intravenous access was established, and endotracheal intubation was performed on-site. She was transported to a local hospital for initial evaluation. At the referring hospital, contrast-enhanced computed tomography revealed a 20-cm rupture of the left diaphragm with gastric herniation, spleen injury, multiple left rib fractures, and burst fractures of the L3 to L4 vertebrae (Fig. 1A–D). Despite fluid resuscitation, her hemodynamic instability persisted. Owing to the lack of immediate surgical capacity, the patient was transferred to our institution. The transport from the referring hospital to our facility took approximately 20 minutes.

**Figure 2.**
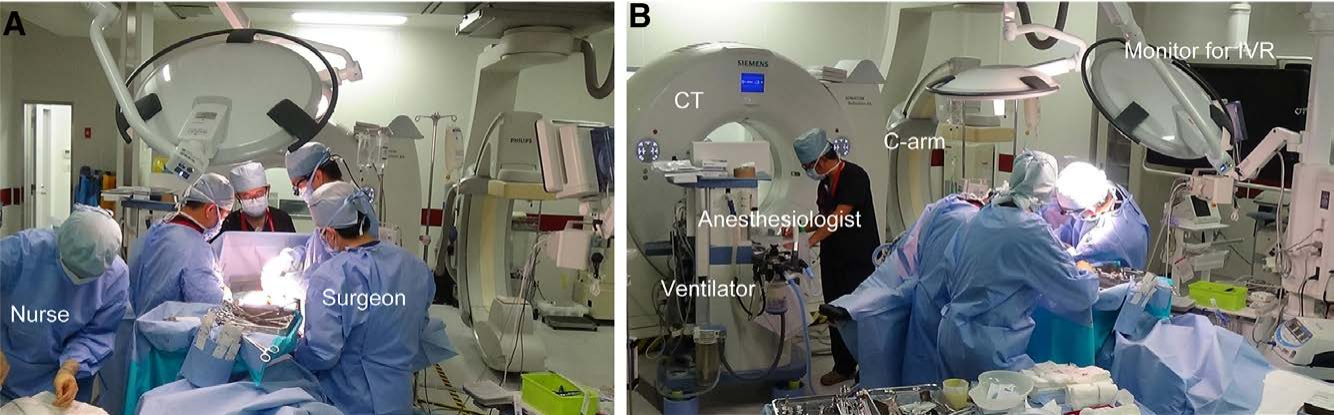
Hybrid emergency room system at the time of emergency surgery (A and B). CT = computed tomography, IVR = interventional radiology.

Upon notification, our HERS was activated. The HERS is equipped with a CT scanner, anesthesia setup, fluoroscopic imaging, and an integrated operating theater (Fig. 2). Based on pre-arrival information, the multidisciplinary trauma team assembled in HERS prior to the patient’s arrival. Utilizing a shared visual planning board, team members collaboratively organized and shared available information in a chronological sequence. This pre-arrival briefing facilitated a shared understanding of the patient’s condition and enabled the rapid formulation of a tailored treatment strategy. Upon the patient’s arrival, vital signs and imaging were reevaluated, followed by a brief multidisciplinary discussion. Consequently, an emergency laparotomy was initiated 26 minutes after arrival. Upon arrival, the patient was receiving noradrenaline at 0.2 μg/kg/min, with a blood pressure of 117/98 mm Hg, heart rate of 142 bpm, respiratory rate of 36 breaths/min, and Glasgow Coma Scale score of E1V1M1 (total score: 3). Detailed neurological assessment was not possible due to deep impaired consciousness; no purposeful movement was observed even to painful stimuli. Laboratory analysis on arrival revealed a hemoglobin level of 14.0 g/dL and platelet count of 1,76,000 cells/μL. The patient’s prothrombin time was 16.4 seconds, and her fibrinogen level was 88 mg/dL. Arterial blood gas analysis revealed a pH of 7.210, base excess of −7.3 mmol/L, and lactate level of 4.0 mmol/L. Her injury severity score was 26, the revised trauma score was 3.80, and the calculated probability of survival was 0.216.^[6,7]^ A midline laparotomy revealed a 20-cm rupture of the left diaphragm with herniation of the stomach into the left thoracic cavity. Superficial splenic bleeding was identified and controlled via direct pressure and electrocautery. No injury to the lungs, great vessels, or chest wall was observed during the transdiaphragmatic inspection. The diaphragmatic defect was repaired using continuous sutures.

The flail chest was managed postoperatively with mechanical ventilation. Management in the intensive care unit primarily focused on ventilatory support for the flail chest.

A staged surgical strategy was adopted. On hospital day 9, internal fixation with plates was performed for the left rib fractures. On day 10, posterior spinal fixation was performed for the L3 to L4 burst fractures. These subsequent surgeries were performed in a conventional operating room.

**Figure 3.**
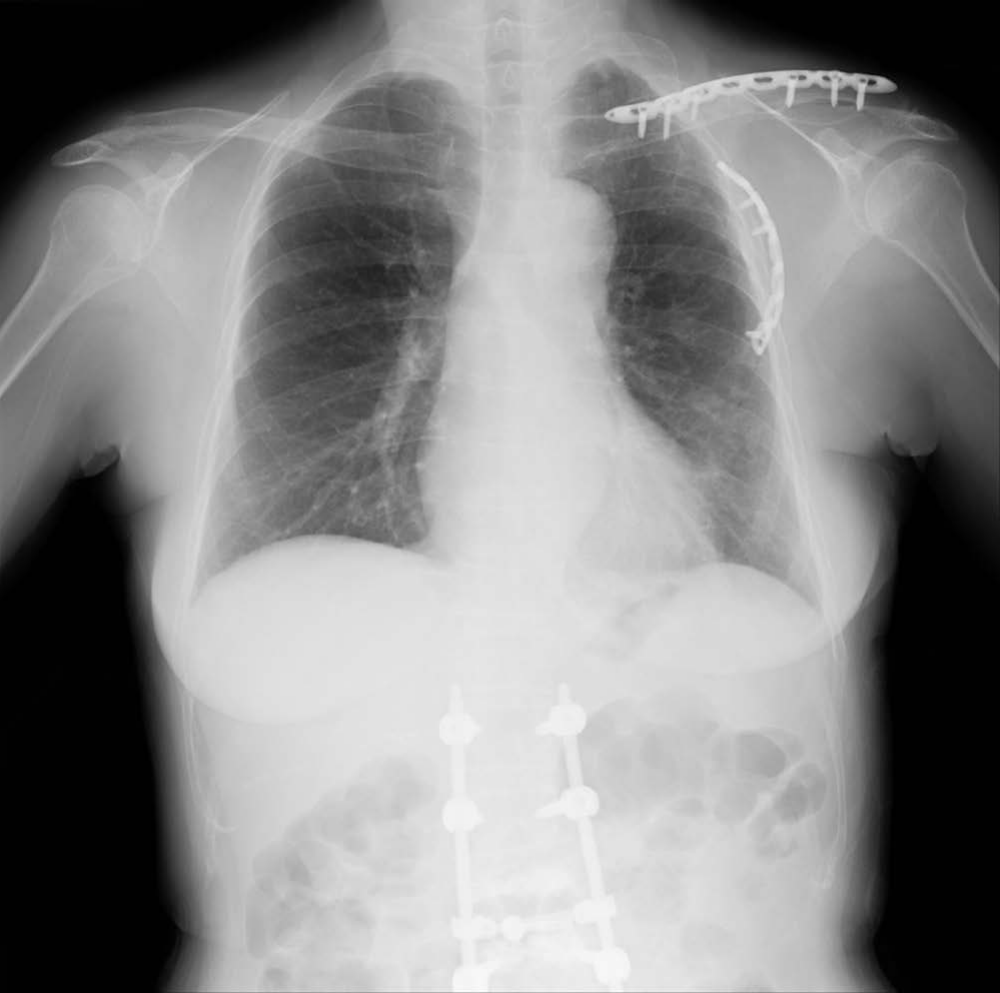
Chest radiograph at discharge shows good general condition of the chest wall, clavicle, and lumbar spine.

**Figure 4.**
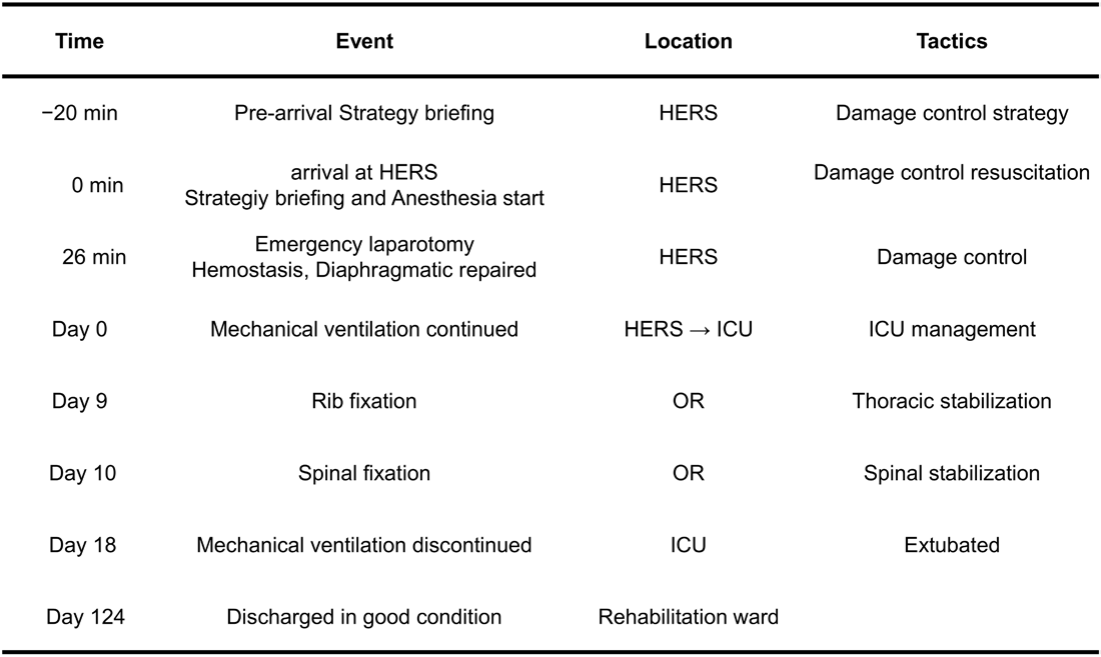
Timeline of key clinical events and interventions. HERS = hybrid emergency room system, ICU = intensive care unit, OR = operating room.

The patient was successfully weaned off mechanical ventilation on hospital day 18. No signs of spinal cord injury were observed. The patient was transferred to a rehabilitation ward for postoperative rehabilitation following spinal surgery and respiratory rehabilitation following rib fixation and discharged in good ambulatory condition on hospital day 124 (Fig. 3). The patient remains neurologically intact and in good health at 3 years post-injury. Written informed consent was obtained from the patient for the publication of this case report. All efforts were made to ensure anonymity. Figure 4 shows the timeline of key events that occurred and the interventions conducted during the clinical course.

## 3. Discussion

This case demonstrates the use of the HERS to initiate a staged damage control strategy in a patient with severe multiple trauma, including a diaphragmatic injury, spleen injury, flail chest, and lumbar spine fractures. Rapid assessment and emergency laparotomy were performed in the HERS, followed by staged definitive surgeries, leading to a favorable clinical outcome. This case highlights the potential role of the HERS in enabling early therapeutic intervention through rapid strategic decision-making and facilitating a structured transition to subsequent staged surgical strategies in the management of severe trauma. Following detailed analysis, 2 key strategic insights were identified.

First, the HERS functioned not merely as a procedural space but as a platform facilitating early strategic decision-making and immediate execution. Pre-arrival information sharing enabled coordinated decision-making upon the patient’s arrival, leading to laparotomy initiation within 26 minutes. Early strategic decision-making is critical for improving trauma outcomes.^[1,2]^ Although no extraordinary technologies were used in this case, gathering key personnel in an integrated space allowed real-time information sharing and rapid strategic consensus. These findings suggest that HERS can support rapid interventions and act as a catalyst for real-time strategic formation and execution. Tools such as centralized monitors, electronic briefing systems, and AI-assisted dashboards can be integrated in the system, as they could enable real-time visualization and provide updated patient information.^[8]^ These technologies have the potential to strengthen the role of HERS in time-sensitive clinical decision-making.

Furthermore, this case demonstrates how the initial damage control strategy was effectively extended into a staged surgical approach based on the patient’s physiological condition. Recognizing the risk of ventilatory instability associated with prone positioning for spinal fixation in a patient with flail chest, the surgical team prioritized rib fixation to stabilize respiratory function before proceeding with spinal surgery. This surgical sequence adhered to the principles of the initial damage control strategy, allowing for safe ventilation management and staged surgical intervention. Although earlier rib fixation may have facilitated earlier weaning from mechanical ventilation, the adopted staged approach ultimately resulted in successful ventilator weaning and discharge without neurological deficits. These findings are consistent with those of previous reports supporting early chest wall stabilization in patients with a flail chest,^[9]^ further reinforcing the importance of staged surgical management as an extension of the damage control strategy.^[2]^

Implementing the HERS in clinical settings presents several challenges. Physical infrastructure alone is not sufficient; successful operation requires a comprehensive institutional framework centered around HERS, smooth interdepartmental coordination, and round-the-clock availability of surgical and critical care services.^[5,8]^ In the present case, HERS may have facilitated early strategic decision-making, resulting in a favorable clinical outcome. Based on our experience, we believe that insufficient pre-arrival information can delay trauma team activation and hinder timely decision-making. This case highlights how efficient sharing of pre-arrival information and a well-coordinated workflow can maximize the benefits of HERS usage. Although not yet widely implemented, certain emerging technologies, such as AI-assisted triage, 5G communication, and telemedicine, could be actively integrated into the HERS framework to help address its limitations^[5,8,10]^; this integration may enhance coordination, expedite strategic decision-making, and facilitate broader adoption. Moreover, the resulting advanced and integrated form of HERS could reinforce the role of HERS not only as a procedural space but also as a foundation for early trauma strategy.^[5,8]^

HERS served as a procedural space as well as a platform for early strategic decision-making, enabling timely intervention through real-time information sharing within an integrated multidisciplinary environment. However, one must note that this report is based on a single case, and its generalizability is limited. Nonetheless, barriers to implementation, such as staffing, infrastructure, and system integration, remain substantial.^[3–5,8]^ Future studies should focus on assessing the cost-effectiveness of HERS and on developing certain standardized frameworks that can facilitate a broader adoption of HERS.

## 4. Conclusion

This case illustrates that damage control strategy encompasses not only rapid initial surgical intervention but also early strategic decision-making and staged surgical management. The HERS facilitated real-time information sharing and coordinated execution, potentially contributing to favorable outcomes. Emphasizing early strategic planning within such integrated systems may be crucial for optimizing trauma care in the future.

## Author contributions

**Conceptualization:** Teppei Tokumaru.

**Data curation:** Hideaki Kurata, Michiaki Hata, Joji Tomioka.

**Formal analysis:** Teppei Tokumaru.

**Investigation:** Teppei Tokumaru, Hideaki Kurata, Michiaki Hata, Joji Tomioka.

**Supervision:** Takehiro Okabayashi, Yuichi Saisaka.

**Visualization:** Teppei Tokumaru.

**Writing – original draft:** Teppei Tokumaru.

**Writing – review & editing:** Teppei Tokumaru, Takehiro Okabayashi, Joji Tomioka.
